# Genomic responses in rat cerebral cortex after traumatic brain injury

**DOI:** 10.1186/1471-2202-6-69

**Published:** 2005-11-30

**Authors:** Christina von Gertten, Amilcar Flores Morales, Staffan Holmin, Tiit Mathiesen, Ann-Christin Sandberg Nordqvist

**Affiliations:** 1 Department of Clinical Neuroscience, Karolinska Institutet, Section of Clinical CNS research, Karolinska University Hospital, SE-171 76 Stockholm, Sweden; 2 Department of Molecular Medicine, Karolinska Institutet, Stockholm, Sweden

## Abstract

**Background:**

Traumatic brain injury (TBI) initiates a complex sequence of destructive and neuroprotective cellular responses. The initial mechanical injury is followed by an extended time period of secondary brain damage. Due to the complicated pathological picture a better understanding of the molecular events occurring during this secondary phase of injury is needed. This study was aimed at analysing gene expression patterns following cerebral cortical contusion in rat using high throughput microarray technology with the goal of identifying genes involved in an early and in a more delayed phase of trauma, as genomic responses behind secondary mechanisms likely are time-dependent.

**Results:**

Among the upregulated genes 1 day post injury, were transcription factors and genes involved in metabolism, e.g. STAT-3, C/EBP-δ and cytochrome p450. At 4 days post injury we observed increased gene expression of inflammatory factors, proteases and their inhibitors, like cathepsins, α-2-macroglobulin and C1q. Notably, genes with biological function clustered to immune response were significantly upregulated 4 days after injury, which was not found following 1 day. Osteopontin and one of its receptors, CD-44, were both upregulated showing a local mRNA- and immunoreactivity pattern in and around the injury site. Fewer genes had decreased expression both 1 and 4 days post injury and included genes implicated in transport, metabolism, signalling, and extra cellular matrix formation, e.g. vitronectin, neuroserpin and angiotensinogen.

**Conclusion:**

The different patterns of gene expression, with little overlap in genes, 1 and 4 days post injury showed time dependence in genomic responses to trauma. An early induction of factors involved in transcription could lead to the later inflammatory response with strongly upregulated CD-44 and osteopontin expression. An increased knowledge of genes regulating the pathological mechanisms in trauma will help to find future treatment targets. Since trauma is a risk factor for development of neurodegenerative disease, this knowledge may also reduce late negative effects.

## Background

Traumatic brain injury (TBI) is a complex disorder, often with a severe or deadly outcome. The primary phase with tissue disruption initiates secondary injury processes in a delayed phase causing pathophysiological changes in the brain. As a consequence of the initial mechanical impact to the head, cerebral metabolism, blood flow and ion homeostasis are altered for a period of hours to days and even months [1]. During the secondary injury high levels of glutamate, Ca^2+ ^and lactate are released, and cytokines are produced, leading to an inflammatory response, which all contribute to further tissue damage [2]. Beside the destructive processes, endogenous neuroprotective events in repair and regeneration also occur [3]. However, harmful processes dominate and eventually lead to tissue loss due to cell death. A challenge lies in understanding the molecular mechanisms behind the pathological processes, and the complex interplay between the different genes and their pathways operating after TBI. Despite a growing literature and extensive research on TBI, current clinical treatments are insufficient to support the repair processes and obstruct secondary injuries why preventive measures might be the most efficient way to improve outcomes.

The disrupted homeostasis in energy and oxygen supply leads to activation of several systems. Transcription, growth, plasticity, differentiation, signalling, inflammation, and cell death genes are affected in different trauma models. The injury alters mRNA and/ or protein levels for e.g. growth factors like NGF, BDNF, and the IGF system [4,5], apoptotic and anti-apoptotic factors like caspases, bax and bcl-2 [6,7], immediate early genes such as c-fos, c-jun and JunB [8], inflammatory markers like interleukines [9] and heat shock proteins [10].

The microarray technique gives an opportunity to simultaneously look at gene expression changes in a large number of genes. It is thus a means to cope with multiple processes, which may well be a prerequisite to handle the complexity of TBI. Trauma reports in mice and rat have mainly focused on early time points around 2 – 72 hours after trauma [11-17]. Later time-points after TBI have not been studied in the rat, although late changes in mice show similarities to those induced by ischaemia in rats [18]. Genes operating at later time-points may well be linked to the continuous brain tissue damage and secondary injuries, which develops after impact. As molecular responses to trauma are time-dependent, we have compared RNA expression after TBI in individual rats 1 and 4 days post injury (dpi). We used the weight-drop technique to produce a cerebral cortical contusion (CCC) [19], which mimics the clinical situation of focal contusion in patients. Alterations in RNA levels in the injured cortex were compared to the uninjured cortex and analysed with a cDNA microarray containing 6200 probes. We found more affected genes 1 dpi than 4 dpi with little overlap existing between the two time points. Significant differences between 1 and 4 dpi were seen in groups of upregulated genes involved in e.g. transcription, metabolism and cell proliferation. Genes involved in proteolysis and immune responses were significantly overrepresented in the delayed phase, which probably are important in secondary injuries.

## Results

### cDNA microarray

**Figure 1:**
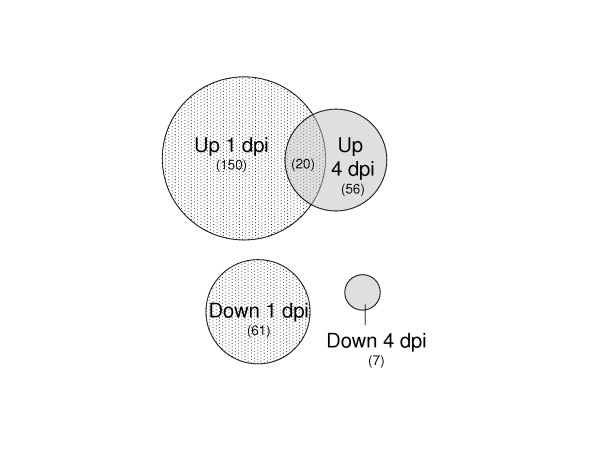
**Venn diagram illustrating regulated genes after cerebral cortical contusion 1 and 4 dpi. **Overlapping area shows genes for which expression was altered at both time points. Number of regulated genes is noted in parenthesis.

A cDNA microarray containing 6200 gene probes was used to study changes in gene expression induced by CCC in the ipsilateral cortex (including site of injury) in comparison to the contralateral uninjured cortex. At 1 dpi, the expression of 150 genes was significantly increased [see Additional file 1] while 61 genes were downregulated [see Additional file 2]. In contrast, 4 dpi displayed 56 upregulated genes [see Additional file 1], while 7 were downregulated [see Additional file 2]. Only 20 genes were upregulated both 1 and 4 dpi, while none of the downregulated genes were in common for the two time points (Fig. 1).

**Figure 2:**
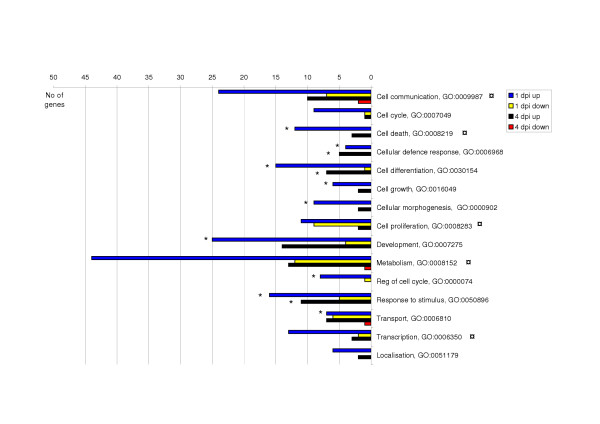
**Functional classification of regulated genes 1 and 4 days after cerebral cortical contusion. **Up- and downregulated genes with fold changes >1.6 at a 2% false discovery rate, annotated and categorised by the gene ontology database eGOn. Note a significant (p < 0.05) overrepresentation of upregulated genes in relation to total number of expressed genes (*) in the functional groups of cell differentiation, defence response and response to stimulus, for both 1 and 4 days post injury (dpi), while 1 dpi also showed significance (p < 0.05) for genes involved in cell death, cell growth, cellular morphogenesis, development, regulation of cell cycle and transport. Additionally, genes with increased expression showed significant difference (p < 0.05) between 1 and 4 dpi in the groups of cell communication, cell death, cell proliferation, metabolism and transcription (¤) analysed by eGOn.

The genes were grouped in functional categories using the functional classification proposed by the Gene Ontology Consortia, to allow for analysis of the response 1 and 4 dpi. A comparison of frequency distribution between gene ontology (GO) categories related to biological function, between the whole set of expressed genes and upregulated genes (Fig. 2), showed that both 1 and 4 dpi displayed a significant overrepresentation (p < 0.05) of upregulated genes in the categories 'cell differentiation' (GO:0030154), 'cellular defence response' (GO:0006968) and 'response to stimuli' (GO:0050896). Additionally at 1 dpi, 'cell growth' (GO:0016049), 'transport' (GO:0006810), 'development' (GO:0007275), 'cell death' (GO:0008219) and 'regulation of cell cycle' (GO:0000074) showed significant upregulation. Four days post injury, significant upregulation of 'immune response' (GO:0006955) and 'proteolysis and peptidolysis' (GO:0006508) were observed. Comparing the groups of downregulated genes to the whole set of expressed genes resulted in no significant category with the frequency distribution test, neither for 1 dpi nor 4 dpi. Other regulated genes were found in most functional categories indicating that response to injury is complex and affects many different biological processes.

Next, we analysed if there were statistically significant differences between the distributions of genes in functional categories when comparing 1 and 4 dpi (Fig. 2). Significantly (p < 0.05) different distribution of upregulated genes among GO categories were detected for 'cell communication' (GO:0009987), 'cell death' (GO:0008219), 'cell proliferation' (GO:0008283), 'metabolism' (GO:0008152) and 'transcription' (GO:0006350) clearly indicating the qualitative differences in response to trauma at the two different time points. There were no statistical differences in the GO categories between downregulated genes comparing 1 and 4 dpi. However, only seven genes were found to be downregulated at 4 dpi.

Regulated genes were also grouped manually, without taking multiple functions for a gene in consideration. 'Transcription and translation' was a large group at 1 dpi, whereas at 4 dpi genes in the groups 'proteases and their inhibitors' and 'ECM (extra cellular matrix) and cytoskeleton' were well represented [see Additional file 1]. Among regulated genes with decreased expression, 'transporters, channels and binding proteins' and 'metabolism' were large groups at day 1 [see Additional file 2].

### PCR, in situ hybridisation and immunohistochemistry

**Figure 3:**
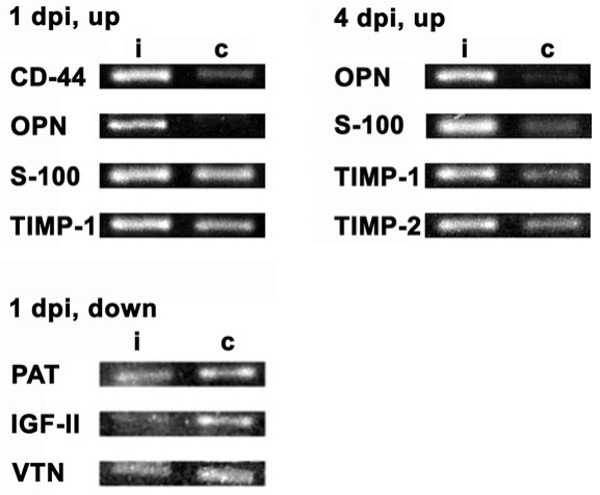
**Confirmation of regulated genes after microarray analysis. **Genes with altered expression analysed by reverse transcriptase-polymerase chain reaction (RT-PCR), comparing ipsilateral (i) to contralateral (c) hemisphere. OPN – osteopontin, TIMP-1/-2 – tissue inhibitor of matrix metalloproteinase, PAT – angiotensinogen, IGF-II – insulin like growth factor – II, and VTN – vitronectin.

**Figure 4:**
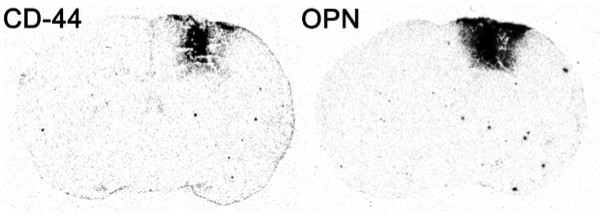
**Localisation of CD-44 and osteopontin mRNA expression. **Autoradiographs following *in situ *hybridisation of rat brain sections after contusion showing CD-44 and OPN – osteopontin mRNA expression. Note intense mRNA signal in contusion area with no visible signal in contralateral hemisphere.

**Figure 5:**
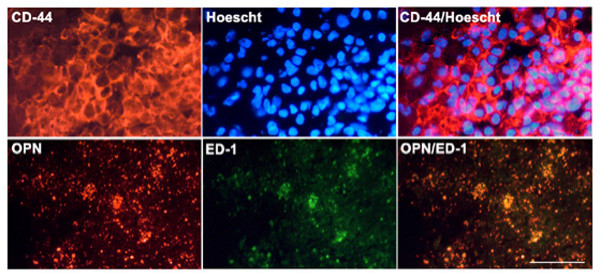
**Fluorescence photomicrographs showing CD-44 and osteopontin immunoreactivity, 4 dpi. **High-power photomicrographs of injured rat brains close to impact show immunofluorescence staining for CD-44 and osteopontin (OPN). Nuclear staining with Hoescht illustrates, in the merged picture, (CD-44/Hoescht) the cytoplasmatic staining for CD-44. Immunofluorescence staining for ED-1, a macrophage marker, demonstrates, in the merged picture, (OPN/ED-1) co-localisation with osteopontin. Scale bar = 50 μm.

CD-44, osteopontin (Opn), TIMP-1 and -2 (tissue inhibitors of matrix metalloproteinases), S-100 (injury marker), angiotensinogen (precursor for angiotensin), insulin-like growth factor (IGF)-II, and vitronectin were further analysed by PCR confirming genes as differentially expressed, in line with the microarray results (Fig. 3). Among the set of regulated genes, Opn and CD-44 have potentially important regulatory roles in brain injury. CD-44 is a receptor for Opn and interestingly, CD-44-/- mice are protected from ischaemia induced injury [20]. The fact that both ligand and receptor are upregulated suggests a possible auto-/paracrine regulating loop at the site of the injury involving these genes. To further explore these findings, the expression of Opn and CD-44 were studied by *in situ *hybridisation and immunohistochemistry. Opn mRNA expression was confined to the injury site (Fig. 4) at both 1 and 4 dpi consistent with the microarray result. Its immunoreactivity was co-localised to ED-1, a marker for macrophages and activated microglia (Fig. 5). CD-44 showed a similar local mRNA expression pattern in the damaged region (Fig. 4) with a matching immunoreactivity (Fig. 5) 4 dpi. Nuclear staining with Hoescht showed cytoplasmatic CD-44 expression. Neither *in situ *hybridisation nor immunohistochemistry revealed contralateral expression for either CD-44 or Opn.

## Discussion

In this study we have identified 211 genes that were regulated at 1 dpi and 63 at 4 dpi following a mild focal experimental contusion. The changes at 1 dpi were either initiated by traumatic events that affected gene activation directly, such as depolarisation and increase of intracellular calcium, or as a reaction to traumatically disrupted membranes, ischaemia and increased metabolic demands. At the early time-point, many genes involved in metabolism were affected, such as lactate dehydrogenase and PDH phosphate, which would increase the rate of pyruvate utilisation. Likewise, genes involved in cell growth, cellular morphogenesis, development, transport, cell differentiation, cellular defence response and regulation of cell cycle were significantly upregulated. This was also the case for 'response to stimulus' genes, confirming TBI as a strong inducer of genomic responses. Genes in this group included early mediators such as hsp, Nfkb1, p53; many of which have been identified upregulated previously in experimental trauma [21]. Activation of these genes agrees well with the prevailing theories of response to trauma: ictus leads to activation of reactions that require energy while mitochondrial dysfunction may limit aerobic metabolism.

It is maybe not surprising that more genes were regulated at day 1 than day 4 after injury. Several of the early responding genes were transcription factors with potential to initiate further gene regulation. It is probable that early responses were, to some extent, chaotic while 4 dpi findings represented a situation with less ongoing reparative and/or destructive cellular processes. It may be futile to search for specific destructive pathways with a hope to inhibit these in the early stages: broad gene activation may be non-specific and reflect that the trauma-energy has overcome the activation energy requirements for many different reactions. Our experimental injury model, CCC, creates a focal contusion lesion but does not cause major neurological deficits, prolonged unconsciousness or death, and is therefore considered a mild injury. The experimental injury resembles clinical contusions in patients who have a favourable condition early after injury, but are at risk of deterioration from progression of the focal contusions. This is a typical cause of patients who "talk and die" [22], where energy transfer at the impact has not been sufficient to destroy main vital functions of the brain, but has initiated reactions that destroy neural function in a delayed manner. This is in line with the observation that genes with a role in cell death were markedly overrepresented at 1 dpi and differed significantly from 4 dpi, which could initiate neuronal apoptosis that occurs for a prolonged time after injury in this model [7].

Our study showed significant differences between 1 and 4 dpi for genes with increased expression involved in cell communication, cell proliferation, metabolism and transcription. However, similarly to 1 dpi, 4 dpi also displayed significant upregulation of genes involved in cellular differentiation and response to stimuli. An analysis of the stimulus response 4 dpi showed significance in immune response, which was not present at 1 dpi. The view of the brain as immunologically privileged has been re-evaluated during the last years. Neuroinflammation, either acute in trauma or chronic in neurodegenerative diseases, has been revealed as a pathological mechanism [23]. The defence response in TBI could generate chronic harmful stimuli resulting in neurodegeneration, while it also paves the way for repair. The immune response is likely to be involved in the secondary injury mechanisms working at the later time point.

Opn, strongly upregulated both 1 and 4 dpi, is involved in inflammation, formation of the ECM and cell-matrix interactions. Opn modulates processes like mitochondrial respiration [24] and nitric oxide inhibition [25], affected in TBI. The diverse roles of Opn depend on receptor interaction and phosphorylation state [26]. Opn actions are mediated by integrins and CD-44, a cell surface glycoprotein. Interestingly, CD-44 also showed increased expression levels at 4 dpi. As both Opn and CD-44 are involved in inflammation and neurodegeneration [27] we chose to study these molecules in more detail. Opn mRNA expression and immunoreactivity were confined to the injury site, which co-localised with the macrophage and activated microglia marker ED-1. Opn can induce migration of astrocytes *in vitro*, and has therefore been proposed as an "astrokine" [28]. In our model this could mean that macrophages attract astrocytes to the damaged area, which would be in line with the above proposition. Furthermore, Opn may contribute to the nonpermissive adult CNS milieu, in the glial scar, and inhibiting axon outgrowth [29]. CD-44 mRNA was found locally around and in the lesion area 1 and 4 dpi. This has not been reported previously in this experimental contusion model, but agrees with CD-44 expression after cortical incision in mice [30]. We saw a corresponding immunoreactivity, resembling Opn immunoreactivity, in the penumbra and injured area, indicating interactions between Opn and CD-44. Our findings corroborate studies of upregulated Opn and CD-44 expression in other brain injury models [12,31]. Opn administration was recently reported to be neuroprotective in stroke [32] whereas increased CD-44 mRNA seems to be harmful in ischaemia [20]. It is possible that Opn interacting with CD-44 could activate a pathway leading to further injury, while Opn-integrin interactions would confer neuroprotection. However, other genes affected following injury may also influence the result of Opn expression.

Wound healing in the brain as in other injured tissues requires synthesis of new ECM molecules. The upregulation of fibronectin and collagen expression at 4 dpi could reflect a temporary state with a new matrix, attempting to create an environment more suitable for cell migration. However, we also observed downregulation of vitronectin expression. Vitronectin is an extracellular glycoprotein [33], which could impair cell migration and attachment, and may be necessary for a favourable cell environment. ECM has more functions than mere structural support. For many cell types, ECM acts as a survival factor as cells deprived of matrix attachment are prone to die by apoptosis, and a distorted matrix network also may hinder actions of trophic factors due to defective presentation. Cell attachment generates intracellular signals ending in regulation of cytoskeletal organisation and gene expression [34]. The bridging molecules ezrin and moesin, which were upregulated 1 dpi, and 1 and 4 dpi respectively, probably influence extracellular signals to the cytoskeleton. A way to maintain homeostasis by stabilising the cytoskeleton, might be upregulation of the intermediate filament vimentin (here increased 1 and 4 dpi).

Proteolytic enzymes cause structural ECM changes. Here, CCC altered the expression of many genes for matrix- and matrix modelling molecules reflected in the prominent category 'proteolysis and peptidolysis' specifically at 4 dpi. In this category we found cathepsins but also proteases such as myelencephalon specific protease, which, to our knowledge, is described regulated in this experimental model for the first time. Though proteases are required for re-organisation and plasticity in order to break down obstructing debris, there might be an imbalance in expression between them and their inhibitors, like upregulation of TIMP-1 and TIMP-2 mRNA, or downregulation of neuroserpin. Proteases challenge blood brain barrier (BBB) function. The upregulated MMP-9 (matrix metalloproteinase), involved in BBB breakdown [35], is one marker of secondary injury processes. Further on, the precursor of angiotensin, angiotensinogen, which is involved in BBB reconstitution, was here downregulated 1 dpi [36].

Our experimental contusion resulted in altered mRNA levels for genes in the category 'transcription response' with significant differences between 1 and 4 dpi. Many genes, among them ATF (activating transcription factor)-4, rad (ras associated with diabetes) and retinoic acid receptor, were upregulated at the early time-point, while only a few genes remained altered at the later phase. A novel finding here was the increased expression at 1 dpi of CCAAT/enhancer binding protein (C/EBP)-δ belonging to C/EBP (bZIP) family, which couples extracellular signal transduction pathways to numerous cellular processes and is a potential tumour suppressor gene [37]. Activated C/EBP-δ regulates the neuroprotective IGF-1 [38] and neurotoxic iNOS expression [39], both affected by TBI. Interestingly, another family member, C/EBP-β, is involved in brain injury [40] and neuronal survival [41]. Moreover, C/EBP-β and -δ expression are upregulated by inflammatory stimuli. It is therefore possible that C/EBP-δ also would influence neuronal survival. Another regulated transcription factor belonging to the immediate early gene family was v-myc (homologue to c-myc). Myc is a key molecular integrator of cell cycle machinery and metabolism [42], and many of its target genes were seen upregulated after CCC in this study. Prothymosin-α (PT-α) is a c-myc target [43], associated with cell growth, transcription [44] and recently also suggested to be involved in apoptosis [45]. To our knowledge, we here show for the first time that it's expression level is upregulated following trauma. This knowledge combined with the fact that TBI is a risk factor for neurodegernative disorders with excessive cell death suggests that PT-α may well be a potential candidate for further studies with regard to TBI. Recently, PT-α was shown to associate with STAT-3, an acute phase response factor, resulting in nuclear translocation [46]. In this study increased STAT-3 mRNA levels were noted 1 dpi, corroborating other studies [15,29].

Microarray analyses create large amounts of data and would ideally allow understanding of common regulatory pathways. Database developments with possibilities to better compare similarities and differences between various experimental models will be advantageous when forming new hypotheses. Whether the similarities or differences between affected genes turn out to be the most important factors is empirical: trauma needs to be studied from all its angles.

## Conclusion

This study of genomic responses to trauma comparing 1 dpi to 4 dpi showed significant time-dependent differences, such as the early response in the category 'transcription response' and a more delayed response in the category 'immune defence'. The upregulation of CD-44 and Opn, localised to injury site, probably have important roles in the inflammatory process following TBI. Outcome of their interaction needs to be further studied in order to understand beneficial or detrimental roles. Understanding the pathological mechanisms behind secondary insults and the interplay of operating genes, may help to find treatment targets for brain injury and reduce delayed negative effects, such as neurodegenerative diseases.

## Methods

### Experimental injury model

All studies were conducted in accordance with the guidelines of the regional ethics committee for animal research at the Karolinska University Hospital, Stockholm, Sweden. Totally, 15 male Sprague Dawley rats (BW 250 g; B&K Universal AB, Stockholm, Sweden) were included in the study. Before surgery all rats were anaesthetised with an intramuscular injection of 0.2 ml Hypnorm™ -Dormikum (1:1:2; Hypnorm™ (fentanyl citrate 0.315 mg/ml, fluanisone 10 mg/ml, Janssen Pharmaceutica, Beerse, Belgium): Dormikum (1 mg/ml, Roche AB, Stockholm, Sweden): dH_2_O). Prior to the skin incision, 0.1 ml Xylocaine^® ^(5 mg/ml, Astra, Södertälje, Sweden) was injected subcutaneously in the sagittal midline of the skull and the rats were placed in a stereotactic frame.

### Cerebral cortical contusion

Contusions were performed on 13 rats using the weight-drop model described by Feeney *et al*. [19]. In brief, a craniotomy was made 2.5 mm posterior and 2.5 mm lateral to bregma, and a footplate was placed so that it rested upon the surface of the dura. A stainless tube guided a 24 g weight, which was dropped from a height of 9.3 cm, compressing the tissue at a maximum of 3 mm. After impact, the scalp was sutured and the animals were allowed to recover. Sham operation, craniotomy without contusion, was performed on two animals. The rats were sacrificed at one day (n = 6) and four days (n = 9) by decapitation in Hypnorm™ anaesthesia. Brains (1 dpi n = 3; 4 dpi n = 4) were removed, and impact area with surrounding cortex, ipsilateral and contralateral was dissected out, before quickly frozen in isopentan containing dry ice, prior to RNA isolation. The remaining brains (1dpi n = 3, 4 dpi n= 3, sham n = 2) were removed for sectioning and directly frozen in isopentan – dry ice. Coronal 14 μm cryosections were cut through the center of the impact using a Leica cryostat (CM 3000, Leica Instruments GmbH, Nussloch, Germany). The sections were thaw-mounted onto Super Frost/Plus™ object glasses (Menzel-Gläser, Braunschweig, Germany) and stored at -20°C prior to use.

### RNA extraction

Total RNA was isolated using RNeasy Qiagen kit (VWR International AB, Stockholm, Sweden) according to manufacturer's protocol. Brain tissue was homogenised using a polytron. The RNA was dissolved in diethyl pyrocarbonate (DEPC) treated dH_2_O and quantified by spectrophotometry at A_260 _and A_280_. Quality was verified on a 3-(N-morpholino)-propanesulfonic acid (MOPS)-formaldehyde-agarose gel.

### cDNA microarrays

The cDNA microarrays were described earlier [47] but now extended to comprise about 6200 clones. Clones were selected from the TIGR Rat gene Index [48], Research Genetics [49] and from the lab obtained during differential cloning experiments. Arrays were pre-hybridised in 1% bovine serum albumin (BSA), 5 × SSC (1 × SSC: 0.15 M NaCl, 0.015 M sodium citrate) and 0.1% sodium dodecyl sulfate (SDS), at 42°C for 1–2 hours, washed in milli-Q H_2_O and dried immediately before the probe was applied.

### cDNA labelling, purification, and hybridisation

Total RNA (30 μg) was used from individual animals in each hybridisation. Labelled cDNA was produced using an oligo-dT primer and Cy3-/ Cy5-uridine 5'-triphosphate labelled nucleotides (PerkinElmer, MA) in reverse transcription using Superscript II (Life Technologies Inc., NY). Cy3- and Cy5-labelled cDNAs were pooled and purified using Microcon 30 columns (Millipore, MA) and then adjusted to a final volume of 25 μl with hybridisation buffer [3.4 × SSC, 0.3% SDS, 20 μg mouse Cot-1 DNA (Invitrogen, CA), 20 μg poly A RNA, 20 μg yeast tRNA]. After heating at 98°C for 2 min and cooling to room temperature, the probe was added to the array and covered by a plastic cover-slip, put in a sealed hybridisation chamber (Corning Inc., NY), and hybridised at 65°C for 15–18 hours. Then the array was washed, dried and immediately scanned with a GMS 418 scanner (Affymetrix, CA). Analyses were made on individual animals and with dye-swap to account for dye-biased effects; 1 dpi n= 3 (two animals with ipsilateral total RNA labelled with Cy5 and one animal with ipsilateral total RNA labelled with Cy3) and 4 dpi n= 4 (two animals with ipsilateral total RNA labelled with Cy5 and two with ipsilateral total RNA labelled with Cy3).

### Data processing and analysis

Image analysis was performed with GenePix Pro software (Axon instruments, CA). Automatic and manual flagging were used to localise absent or very weak spots (< 2 times above background), which were excluded from analysis. The signal from each spot was calculated as the average intensity minus the average local background. Expression ratios of Cy5/Cy3 (or Cy3/Cy5 in case of dye-swap) were normalised using a method that takes into account and corrects for intensity-dependent artefacts in the measurements; the locally weighted linear regression (Lowess) method in the SMA package (Statistics for Microarray Analysis)[50,51]. SMA is an add-on library written in the public domain statistical language R.

The significance of expression ratios was statistically evaluated using the SAM (Significance Analysis of Microarrays) technique [52]. Similarly to the familiar *p*-value, a *q*-value was assigned each of the detectable genes in the array. The q-value measures the lowest false discovery rate (FDR) at which the gene is called significant. A 2% FDR was used to identify regulated genes. On top of the SAM criteria, a mean ratio cut off (log2 ratio injured/uninjured > 0.7 corresponding to > 1.6-fold regulation) was applied to describe ratios as up-/down-regulated and was applied to each hybridised microarray. Using these settings one could expect three genes falsely identified as regulated among the 150 upregulated 1 dpi and one gene among those identified 4 dpi. Data has been deposited in Gene Expression Omnibus [53].

We used the web based tool eGOn v 1.0 (explore Gene Ontology, developed at the Norwegian University of Science and Technology) [54] to functionally classify the transcripts. A total of 3414 genes 1 dpi and 2242 genes 4 dpi were deposited in eGOn of which 1272 genes for 1 dpi and 904 genes for 4 dpi were annotated by eGOn and categorised into gene ontology (GO) categories related to biological function. Each list of differentially expressed genes were compared to all genes expressed, for 1 respectively 4 dpi with the two sided one-sample binomial test implemented in eGOn. Comparison was made to test if the proportion of genes upregulated at 1 dpi was different from the proportion of genes upregulated 4 dpi, in eGOn, which use McNemars test based on an implementation using the binomial distribution. Additionally, also using eGOn, upregulated genes were compared to downregulated genes for 1 and 4 dpi separately with Fishers exact test.

The regulated genes presented in tables 1 and 2 were grouped based on information from Gene ontology, DAVID (Database for Annotation and Visualisation and Integrated Discovery) [55], PubMed and other existing array reports. A gene can be annotated for several functions, which is not displayed in the tables where a gene is assigned to only one group. However, the statistical testing in eGOn has taken multiple functions for genes in consideration, why one and the same gene can be found in several of the categories made by eGOn. Therefore the groups in the tables do not correspond to the groups of biological function made by eGOn.

### RT-PCR

cDNA templates for RT-PCR were generated from five μg of total RNA treated with DNase I (Roche Diagnostics Scandinavia AB, Bromma, Sweden) in a reaction with 1 U DNase I/μg RNA, 25 mM Tris (pH 8.0), 25 mM NaCl, 5 mM MgCl_2 _and 0.15 U rRNasin RNA inhibitor (Promega, Madison, WI) for 25 min at 37°C. Single stranded cDNA synthesis was made using PowerScript Reverse Transcriptase (Clontech, CA) according to manufacturer's protocol. RT-PCR was performed using Taq Dynazyme (Finnzymes, Espoo, Finland) under standard conditions (1 × Dynazyme buffer, 0.2 mM dNTPs (Life Technologies), 0.5 U Taq Dynazyme, 1 μM of each specific primer and 2 μl of cDNA) using a 4-min hot start at 94°C followed by 30 cycles of 94°C for 45 sec, 59°C for 45 sec, 72°C for 1 min, followed by 10 min final extension at 72°C. Glucose-6-phosphate dehydrogenase (G6PD) was co-amplified as an internal control in each reaction. PCR products were analysed by 1.5% agarose gel electrophoresis (Sigma, St Louis, MO), and visualised using ethidium bromide fluorescence. All primer pairs (Table 1) were obtained from MedProbe (MedProbe AS, Oslo, Norway).

### In situ hybridisation

Synthetic oligonucleotide probes (Table 1) were synthesised and purified by reverse phase chromatography by Medprobe. The oligonucleotides were labelled at the 3' end with α-^35^S-dATP ((NEG034H) du Medical NEN, Bruxelles, Belgium) using terminal-deoxynucleotidyl-transferase TdT (Takara, Amersham Pharmacia Biotech, Uppsala, Sweden) at 37°C for 1 hour and purified using mini Quick Spin Oligo Columns (Roche Diagnostics Scandinavia AB). The specific activities obtained ranged from 1–4 × 10^9 ^cpm/μg oligonucleotide. 14 μm sections were air-dried for one hour and covered with a hybridisation buffer containing 50% formamide, 4 × SSC, 1 × Denhardt's solution (0.02% polyvinyl-pyrrolidone, 0.02% bovine serum albumin, and 0.02% Ficoll), 1% sarcosyl, 0.02 M phosphate buffer, 10% dextran sulphate (Amersham Pharmacia Biotech), 500 μg/ml heat-denatured salmon sperm DNA (Sigma) and 200 mM DTT (Amersham Pharmacia Biotech), and 1 × 10^7 ^cpm/ml labelled probe. The slides were incubated in a chamber humidified with 4 × SSC and 50% formamide for 16–20 hours at 42°C. After hybridisation, the sections were rinsed in 1 × SSC at 40°C, 4 × 15 min in 1 × SSC at 55°C, 1 × SSC and dH_2_O, 1 min each at room temperature followed by dehydration with 60% and 95% ethanol. Sections were air-dried and exposed on BioMax MR X-ray film (Eastman Kodak, Rochester, NY) at room temperature for 6 days.

### Immunohistochemistry

Frozen sections were air-dried, rehydrated in 1 × phosphate-buffered saline (PBS) and fixed in 4% buffered paraformaldehyde for 7 min at room temperature, rinsed in 1 × PBS, and blocked with normal goat serum (1:150) in 1% BSA for 30 min at room temperature. Labelling was made overnight with a monoclonal mouse anti rat CD-44 antibody 1:100 (Serotec, Oxford, UK), a mouse monoclonal anti rat MPIIIB10_1 _(osteopontin) antibody 1:50 (Developmental Studies Hybridoma Bank, IA), or mouse anti rat ED-1 antibody 1:1000 (Serotec) at 4°C. Sections were rinsed in 1 × PBS and incubated with fluorescent or indocarbocyanine (Cy3)-conjugated goat anti-mouse 1:1000 (Jackson Immunoresearch Lab. Inc, PA) or fluorescein isothiocyante (FITC)-conjugated goat anti mouse 1:150 (Jackson Immunoresearch Lab.) for 1 hour at room temperature. Nuclear staining with Hoescht was performed on slides with CD-44 immunolabelling. After washing, the slides were mounted with glycerol:PBS. Evaluation of staining was performed by fluorescence microscopy with Leica filter cube N2.1 (excitation filter: 515–560 nm, suppression filter edge wavelength: 590 nm) for detection of Cy3-labelling and Leica filter cube L4 (excitation filter: 450-490 nm, suppression filter edge wavelength: 515–560 nm) for FITC labelling. Photomicrographs for double labelling illustrations were obtained by changing filter cube without altering section position or focus.

## Authors' contributions

SH performed the experimental neurosurgery with assistance of CvG. ACSN and CvG sampled and sectioned the tissue, and performed *in situ *hybridisation. CvG prepared the material and performed the cDNA microarray experiments, RT-PCR, and immunohistochemical stainings. AFM and CvG analysed the microarray data. CvG, AFM, TM, ACSN designed the study and drafted the manuscript. All authors read and approved the final manuscript.

## Supplementary Material

Additional File 1 **Upregulated genes 1 and 4 dpi following cerebral cortical contusion. **The table shows clone identity (Clone ID), accession number (Acc no), gene name, and fold change 1 and 4 days post injury (dpi) after a cerebral cortical contusion (CCC). Bold numbers are regulated genes with fold change > 1.6 at a false discovery rate ≤ 2%. * = similar to, EST = expressed sequence tag. Negative signs denote downregulated fold changes.
Click here for file

Additional File 2 **Downregulated genes 1 and 4 dpi following cerebral cortical contusion. **The table shows clone identity (Clone ID), accession number (Acc no), gene name, and fold change 1 and 4 days post injury (dpi) after a cerebral cortical contusion (CCC). Bold numbers are regulated genes with fold change > 1.6 at a false discovery rate ≤ 2%. * = similar to, EST = expressed sequence tag. Negative signs denote downregulated fold changes.
Click here for file

## Figures and Tables

**Table 1:** Primers and oligonucleotide probes used for confirmation of results.

Gene	Primer/oligonucleotide probe sequence (nts)	Product (bp)	Acc. no
PAT	F: 5'-GAAGCTAGAGGCTGAGGATC- 3' R: 5'-GTGCAGTCTCCCTCCTTCAC- 3'	233	L00091
S-100	F: 5'-GGACCTGAGAGTGCTCATGG- 3' R: 5'-GCATGCAATGATGAGCCCCG-3'	222	J03627.1
IGF-II	F: 5'-GACTGAGTTGGGGCAAATAC- 3' R: 5'-CAGGTGTTAGGAAGGTGCTC- 3'	211	AA899788
CD-44	F: 5'-CCGACCTTCCCACTTCACAG- 3' R: 5'-TCTCCTCGCAGGACCAGAAG- 3' 5'- ATCACTGGTGGCCAGGGTGCTCCCA ATAAAGAAGGCGTCATCCC-3' (44-mer)	200	AA817820
OPN	F: 5'-CTGCCAGCACACAAGCAGAC- 3' R: 5'-ACTCCTTGGACTGCTCCAGG- 3' 5'-TCCTGATCAGAGGGCACGCTCAGAC GCTGGGCAACTGGGATGACC-3' (45-mer)	323	M14656
TIMP-1	F: 5'-CGAGACCACCTTATACCAGCG-3' R: 5'-CAGGAAGCTGCAGGCAGTGAT-3'	216	L31883
TIMP-2	F: 5'-TGCACCCGCAACAGGCGTTTT-3' R: 5'--TTCCTCCAACGTCCAGCGAGA-3'	224	L31884
VTN	F: 5'-ATCGACGCTGCCTTCACTCG-3' R: 5'-TGGCGCCATCAGAGGATCTG-3'	373	U44845

Nts = nucleotides, bp = base pairs, acc. no = accession number
