# Supplementary material for: Genomic responses in rat cerebral cortex after traumatic brain injury
Source: BMC Neurosci. 2005 Nov 30;6:69. doi: 10.1186/1471-2202-6-69 (PMC1310614; doi:10.1186/1471-2202-6-69)
Supplement: Additional File 1 — Upregulated genes 1 and 4 dpi following cerebral cortical contusion. The table shows clone identity (Clone ID), accession number (Acc no), gene name, and fold change 1 and 4 days post injury (dpi) after a cerebral cortical contusion (CCC). Bold numbers are regulated genes with fold change > 1.6 at a false discovery rate ≤ 2%. * = similar to, EST = expressed sequence tag. Negative signs denote downregulated fold changes. [file 1471-2202-6-69-S1.pdf]

Clone ID	Acc no	Gene name	1 dpi, CCC	4 dpi, CCC
**Cytoskeleton, ECM, matrix modelling**				
Common				
RNABT23	AA964431	osteopontin	**11,1**	**56,1**
RGIAC38	AW914230	moesin	**3,3**	**2,7**
RNABM67	AA859385	vimentin	**2,6**	**4,9**
RGIAR28	AF184983	osteoactivin	**2,6**	**10,1**
RGIAN11	AW140624	*fibulin 2	**4,4**	**3,9**
1 dpi				
RGIAE40	BM986315	syndecan 1	**6,7**	3
MKAH 43	M22479	tropomyosin isoform 2	**2,3**	1,7
RNACA35	AA859605	intercellular adhesion molecule-1	**2,1**	-
MKAH 11	D13181	lamin A	**2,1**	1,3
RGIAB8	AW914148	lamin A; lamin C2	**1,6**	-1,2
RNABM48	AA819385	syndecan 4	**2**	-
RNABM92	AA859812	ezrin	**1,8**	1,5
RGIAN83	U06755	acidic calponin	**1,8**	1,6
RGIAK84	NM_012862	matrix Gla protein	**1,7**	1,4
RNACB19	AA957594	EST, fibrillarin	**1,7**	1,3
4 dpi				
RGIAQ36	X15906	fibronectin	1,4	**3,4**
RGIAM23	X70369	collagen, type III, alpha 1	-1,4	**2,1**
RGIAN74	BF281628	collagen type I, alpha-1	-1,3	**1,8**
RGIAQ88	Y13714	osteonectin	-1,2	**1,9**
**Proteases and inhibitors**				
Common				
RGIAQ65	U06179	tissue inhibitor of metalloproteinase-1	**10,4**	**3,2**
RNABP40	AA901070	contrapsin-like protease inhibitor related protein (CPi-26)	**4,2**	**4,9**
RNABL31	AA818798	cathepsin Y	**1,7**	**1,8**
1 dpi				
RNABP80	AA924878	tissue plasminogen activator	**2,9**	1,4
RNABM83	AA900686	endothelin-converting enzyme	**2,1**	1,4
RNABQ32	AA925246	cathepsin K	**1,8**	2,5
RGIAD63	AW914340	sorcin	**1,7**	1,6
RGIAD3	NM_017116	calpain 2	**1,7**	1,3
RGIAO81	AW140681	cystatin beta	**1,6**	1,6
RNABD1	AW917266	carboxypeptidase D	**1,9**	1,3
4 dpi				
RNABM15	AA858673	pancreatic secretory trypsin inhibitor type II	1,5	**3,3**
RGIAB71	AW914199	preprocathepsin D	-	**2,2**
RNABV95	AI058866	tissue inhibitor of metalloproteinases-2	1,7	**2,1**
RGIAD27	X82396	cathepsin B	1,3	**1,8**
RNABV39	AI044130	myelencephalon specific protease	1,4	**1,8**
RGIAX73	AW141002	cathepsin L	1,6	**1,8**
**Metabolism**				
1 dpi				
RNABT53	AA965220	gamma-glutamylcysteine synthetase	**3,9**	-
RNABX41	AI060107	cytochrome P450 1b1	**3,8**	3,9
RGIAF4	M35266	cysteine dioxygenase 1	**2,2**	1,3
RGIAC87	J04791.1	ornithine decarboxylase	**2,2**	-
RGIAN9	NM_017025	lactate dehydrogenase A	**1,8**	1,3
RNABU76	AI029454	phosphofructokinase	**1,7**	1,2
RNABX11	AI058387	pyruvate dehydrogenase phosphatase 1	**1,7**	1,3
RGIAC93	AW914284	*adenylosuccinate synthetase	**1,7**	1,3

RGIAJ85	D17370	CTL target antigen (Cth)/ cystathionine gamma-lyase	**1,7**	-
RNABK11	AA818350	tryptophan-2,3-dioxygenase	**1,7**	-
RNABX26	AI059903	ornithine decarboxylase antizyme inhibitor	**1,6**	-1,1
RNABM26	AA866220	catalase	**2,9**	2,7
RNACD76	AI030897	cytochrome P450, subfamily IID2	**2,1**	1,6
4 dpi				
RGIAR36	AW140788	*glucuronyltransferase I	2,1	**3**
RNABH03	AW141847	spermidine/spermine N1-acetyltransferase		**2**
RGIAD15	BM986384	glutathione reductase	1,3	**1,8**
**Transcription and translation**				
Common				
RNABM55	AA858801	nuclear factor kappa B	**2,2**	**3,4**
RNACB84	AA964516	EST, transforming protein RHOC	**2,5**	**2,2**
1dpi				
RNABR21	AA926277	C/EBP delta	**3,3**	-
RNACF11	AI059251	p55-c-fos proto-oncogene protein	**3,2**	2,1
RNABV84	AI045179	Stat3	**2,9**	-
RNACB54	AA964246	v-myc oncogene homolog	**2,8**	-
RNABY3	AI072276	rad, ras-related protein	**2,8**	-1,2
RNABA73	AW916941	*thioether S-methyltransferase	**2,7**	2,9
RGIAK48	AW140519	c-jun	**2,1**	-
RNACA91	AA957199	EST, serine hydroxymethyltransferase	**1,9**	1,2
RNABQ55	AA926039	EST, cell division control protein CDC21	**1,9**	1,2
RGIAD58	AW914335	cyclin-dependent kinase 4	**1,9**	1,6
RNABS77	AA957218	cyclin D1	**1,8**	1,8
RNACA21	AA858823	activating transcription factor -4	**1,8**	-1,1
RNABX66	AI070100	histone H3.3	**1,7**	1,2
RNACA49	AA859804	EST, eukaryotic translation initiation factor 4e	**1,6**	1,3
RNABK20	AA817843	CCAAT binding transcription factor-B subunit (CBF-A1)	**1,6**	-
RNABN9	AA866415	retinoic acid receptor alpha 2 isoform	**2,9**	-1,1
RNABQ27	AA925012	EST, geminin	**1,6**	1,1
RGIAO87	AW140686	nucleoporin-like protein RAB/Rip	**1,6**	1,2
RGIAN52	BF281613	TIS11	**1,7**	1,1
RNABH17	AW917293	proteasome	**1,7**	-
RNACA38	AA859407	poly A binding protein, cytoplasmic 1	**1,8**	1,7
4dpi				
RGIAG34	AW914509	melanocyte-specific gene 1 protein	3,4	**2,9**
RNACE49	AW916798	mrg1 protein	1,2	**1,9**
RGIAS49	NM_008774	poly A binding protein, cytoplasmic 1	1,7	**1,7**
**Signalling**				
Common				
RGIAP49	L13039	annexin II	**3,3**	**2,5**
RNABU29	NM_010410	orexin precursor	**3,4**	**7,7**
1 dpi				
RNABU33	AA998372	MAP-kinase phosphatase (cpg21)	**4**	1,4
RNABV45	AI045437	neuropeptide Y	**2,7**	2,2
RNABM38	AA858998	RAN, member RAS oncogene family	**2,6**	-
RGIBB95	AW141098	cAMP-specific phosphodiesterase PDE4B	**2,5**	1,2
RNABR42	AA955477	EST, MAP kinase-activated protein kinase 2	**2,1**	1,5
RGIAK19	BI395427	PAK-interacting exchange factor beta2	**1,8**	1,4
MKAE 68	Y08361	RIL	**1,8**	1,5
RNABM47	AA819366	dual-specificity protein tyrosine phosphatase, rVH6	**1,6**	-1,1
RNABV38	AI044049	prepronociceptin	**1,6**	-1
RGIAD7	AW914292	nucleoside diphosphate kinase	**2**	1,4

RNACC37	AA997309	ras-like protein	**1,8**	1,4
4 dpi				
RNABU02	AA998890	annexin III	1,6	**2,1**
RGIAC73	NM_013132	annexin V	1,1	**1,6**
RGIAD87	AW914354	protein tyrosine phosphatase	1,5	**1,6**
**Transporters, channels and binding proteins**				
Common				
RNABO14	AA900235	S-100 related protein	**3,8**	**3,4**
RGIAO59	AF061744	FYN binding protein	**3,7**	**2,8**
RNABR39	NM_145878	fatty acid binding protein 5, epidermal	**1,9**	**2**
1 dpi				
RNABK17	AA817820	CD44	**8,3**	3,2
RNABX10	AI059540	heparin binding epidermal growth factor - like growth factor	**6,8**	-
RGIAD68	AF034607.1	chloride channel ABP	**3,3**	2,4
RNABO13	AA900218	metallothionein	**2,9**	1,9
RNACA59	AA875025	retinoic acid-binding protein I, cellular	**2,5**	-
RNABK89	NM_012588	insulin-like growth factor-binding protein 3	**2,4**	1,7
RGIAK80	M13979	glucose-transporter type 1	**2,3**	1,2
RGIAQ93	AW142622	importin beta-3 subunit	**1,8**	1,6
RNABR75	AA955679	thioredoxin reductase 1	**2,2**	1,2
RNABT27	AA964989	prolyl 4-hydroxylase, beta polypeptide	**2**	1,4
RGIAO42	AW144510	EST, Tax1	**1,8**	1,8
RNACE96	AI058507	*Nedd4 WW binding protein 4	**2,4**	-
4 dpi				
RNABM84	NM_012733	retinol-binding protein 1	2,2	**3,1**
RGIAA74	L01122	ferritin light chain	1,1	**2,1**
RNAA26	X77158	transferrin	-	**1,8**
RGIAJ24	AW142199	*TRAM1	1,4	**2,6**
**Immune system**				
Common				
RNABQ08	AA925356	C1q beta chain	**1,7**	**3,4**
RNABC93	AW917566	class III Fc gamma receptor	**1,8**	**2,3**
1 dpi				
RGIAO1	AW140648	prostaglandin E synthase	**3,5**	1,2
RGIBC53	BF281814	melanoma-associated antigen MG50	**3,5**	2,6
RNACD17	AI028816	natural resistance-associated macrophage protein 2	**1,6**	-1
MKAH 22	K02782	complement component C3	**2**	-
RNACA10	AA819412	interleukin-1 receptor accessory protein	**1,8**	1,3
RNABM5	AA818604	heat shock protein 70	**2,1**	1,3
RNABR40	AA955469	nerve growth factor inducible protein PC4	**2,7**	-1,1
RNABR27	AI146187	MHC class II, DM beta	**2,3**	2,3
4 dpi				
RGIAO94	NM_012488	alpha-2-macroglobulin	1,3	**2,7**
RNABI87	AW916329	beta-2-microglobulin	1,2	**1,6**
RGIAI89	AW140481	CD24 antigen	1,3	**1,8**
RGIAJ90	AW142249	C1q C chain precursor	1,6	**3,2**
RGIAB90	M58405	thymosin beta - 10	1,2	**1,8**
**Growth factors and hormones**				
1 dpi				
RGIAS92	AI070931	bone morphogenetic protein 2	**1,8**	1,8
RGIAF46	AI030286	brain-derived neurotrophic factor	**1,7**	-
RNABU89	AI029586	cholecystokinin (CCK) precursor	**1,8**	1,5
RNACC19	AA996669	epidermal growth factor	**1,6**	1,1
RNABO15	AA899488	transforming growth factor beta -2	**2,1**	1,2

RNABP53	AA924232	*oxytocin	**1,8**	-
**Cell death**				
1 dpi				
RNACF64	AI070487	EST, bcl-2	**1,9**	-1,4
RNABM89	AA875052	p53	**2**	1,1
RNABA90	BF282824	CASP8 and FADD-like apoptosis regulator	**3,2**	1,6
RNABM91	AA875070	prothymosin alpha	**1,6**	1,2
**Cell growth**				
1 dpi				
RNABK65	AA859040	EST, growth arrest and DNA-damage-inducible protein 45	**4,3**	-
RNACB83	AA964601	EST, mitogen inducible gene -2	**1,9**	1,4
RNABT51	AA964194	cell growth regulator rCGR11	**1,7**	1,3
4 dpi				
RGIAA16	BM986218	granulin	-1,2	**2**
**Miscellanous**				
Common				
RNABI21	AW917297	3'(2') 5'-bisphosphate nucleotidase	**8,1**	**94,6**
1 dpi				
RNACE81	AI058471	tissue factor pathway inhibitor precursor	**1,7**	-1,7
RNABU51	NM_016978	ornithine aminotransferase	**1,9**	1,8
RGIAL14	AW142416	*onzin	**2,2**	-
RNABG35	AW915626	AF079530 *Mus musculus syntaxin 13-interacting protein pallid	**1,6**	1,3
RNABC50	AW144061	AK018758 *Mus musculus adult male liver cDNA	**1,7**	1,4
RGIAE37	P34552	C.elegans hypothetical protein R10E12.1	**1,9**	1,1
RGIAQ39	AW142601	ser/thre-like protein lyk4	**2,1**	1,2
RNABN51	BC008272	neuron specific gene family member	**1,6**	1,6
RNABC81	AW917540	chemokine-like factor super family 8	**1,8**	-
RGIAT38	AW913874	EST, adipose differentiation-related protein	**5,7**	2,5
4 dpi				
RGIAD93	BM986275	ER-60 protease	1,4	**1,7**
RNABF77	AW918623	macrophage expressed gene 1	1,4	**1,8**
RNABR08	AA926076	*acid sphingomyelinase-like phosphodiesterase	-1,1	**2**
**ESTs and unknown**				
Common				
RGIAP68	AW140728	unknown	**2,3**	**2,2**
RGIBB88	AW143026	unknown	**1,8**	**1,8**
1 dpi				
RNABQ23	AA924813	EST	**1,7**	1,3
RNABN89	AA899261	EST	**2,2**	1,1
RNACB81	AA964626	EST	**1,8**	1,1
RNABO86	AA923884	EST	**1,7**	-1,1
RNABR18	AA926256	EST	**1,9**	-1,1
RGIAL12	AW142256	unknown	**1,6**	1,2
RGIAM87	AW140618	unknown	**1,8**	3,3
RGIAQ19	AW142367	unknown	**3,4**	1,4
RGIAI91	AW142379	unknown	**2**	1,4
RGIAN35	AW142496	unknown	**2,3**	1,6
RGIAS19	AW142678	unknown	**2,1**	2,2
RGIAS79	AW142703	unknown	**2,4**	1,3
RGIAZ71	AW142932	unknown	**10,1**	5
RGIBC23	AW143043	unknown	**2**	1,2
RNABG5	AW915585	unknown	**2,4**	-
RNABA88	AW916696	unknown	**1,6**	1,6
RNABG53	AW919129	unknown	**2,5**	1,3

RNABH51	AW919155	unknown	**2**	1,3
RNABA87	BF408031	unknown	**1,6**	1,7
RNACD81	AI029644	unknown	**5,1**	2,2
RNACF16	AI059317	unknown	**2,5**	-
RNABG15	AA858655	unknown	**1,9**	-
RNACD58	AI029316	unknown	**1,9**	1
RGIAS57	AA900524	unknown	**2**	1,1
4 dpi				
RNABM16	AA858677	EST	1,3	**2,5**
RNACD54	AI029264	EST	-1,2	**2,3**
RGIBA88	AW142982	unknown	1,3	**1,7**
RNABQ79	AI112925	unknown	-1,2	**2,2**
