# Supplementary material for: Genomic responses in rat cerebral cortex after traumatic brain injury
Source: BMC Neurosci. 2005 Nov 30;6:69. doi: 10.1186/1471-2202-6-69 (PMC1310614; doi:10.1186/1471-2202-6-69)
Supplement: Additional File 2 — Downregulated genes 1 and 4 dpi following cerebral cortical contusion. The table shows clone identity (Clone ID), accession number (Acc no), gene name, and fold change 1 and 4 days post injury (dpi) after a cerebral cortical contusion (CCC). Bold numbers are regulated genes with fold change > 1.6 at a false discovery rate ≤ 2%. * = similar to, EST = expressed sequence tag. Negative signs denote downregulated fold changes. [file 1471-2202-6-69-S2.pdf]

Clone ID	Acc no	Gene name	1 dpi, CCC	4 dpi, CCC
**Cytoskeleton, ECM, matrix modelling**				
1 dpi				
RNABR91	AA956238	vitronectin	**-2,3**	-1,3
4 dpi				
RGIAN17	BF281582	CDCrel-1A	-1,3	**-1,7**
**Proteases and inhibitors**				
1 dpi				
RGIAX36	AW140990	neuroserpin	**-1,8**	-1,4
RGIAB72	X16957	cystatin C	**-1,7**	1,1
RNABQ35	NM_026554	EST, nuclear cap binding protein subunit 2	**-1,9**	-1,4
**Metabolism**				
1 dpi				
RNABT72	AA996833	aldolase C	**-1,7**	-1,4
RNABU17	AA997754	cystathionine beta synthase	**-2,3**	-1,1
RGIAI29	Z36980	D-dopachrome tautomerase	**-1,9**	-1,1
RGIAA64	D00569	2,4-dienoyl-CoA reductase	**-1,6**	1,5
RNABK10	AA818339	glutathione-S-transferase, alpha type	**-1,7**	-1,2
RNABI84	AW916313	glutathione S-transferase, mu type 3	**-1,7**	-1,4
RGIAA23	NM_012598.1	lipoprotein lipase	**-1,6**	-
RGIAI20	M26125	epoxide hydrolase	**-1,9**	-
RGIAE70	U08976	peroxisomal enoyl hydratase-like protein	**-1,6**	1,2
RNABM65	AA859664	1-Cys peroxiredoxin	**-1,6**	-
RGIBB76	AW141091	beta-1,3-glucuronyltransferase 1	**-1,8**	-1,5
RGIAV89	AW140870	putative N-acetyltransferase CML3	**-4,7**	-1,5
RGIAU4	NM_007952	protein disulfide isomerase A3 precursor	**-1,8**	-1,4
RNACA56	AA860063	EST, glutathion S-transferase theta	**-2,2**	-
RNACC94	AA998470	EST, isocitrate dehydrogenase	**-1,6**	1,1
RNACB26	NM_008850	EST, mitochondrial carnitine/acylcarnitine carrier protein	**-1,6**	-1,1
**Transcription and translation**				
1 dpi				
RNABX44	AI059589	D site albumin promoter binding protein	**-2,3**	-1,4
RGIAK8	AW144714	ovalbumin upstream promoter gamma nuclear receptor rCOUPg	**-1,6**	-1,1
**Signalling**				
1 dpi				
RNABI85	AW916314	MAP kinase kinase kinase 1 (MEKK1)	**-1,8**	-1,3
RNABL61	AA819293	soluble guanylyl cyclase alpha 1 subunit	**-2,1**	-1,2
RGIAX10	NM_008708	N-myristoyltransferase 2	**-1,8**	-1,2
RNABY14	AI072201	GABA-A receptor, subunit delta	**-2,2**	-1,8
RGIAW49	NM_008180	Edg-1 orphan receptor 1	**-2,1**	-1,2
RGIBA64	NM_008142	guanine nucleotide binding protein, beta 1	**-1,8**	-1,3
4 dpi				
RNABL4	AA818983	90kDa-diacylglycerol kinase	-	**-1,7**
RNACA4	AA817703	EST, CaM-KII inhibitor	-1,3	**-1,7**
**Transporters, channels and binding proteins**				
1 dpi				
RNABN45	AA900007	phospholemman chloride channel	**-1,9**	1
RGIAV16	AW142770	phospholamban	**-2,4**	-1,2
RNABU18	AA997797	brain digoxin carrier protein	**-2,5**	-1
RNAB 87	S76779	apolipoprotein E	**-1,6**	-
RGIAJ87	AW142246	albumin	**-2,4**	-1,2
RGIAQ87	NM_013930	lysine-ketoglutarate reductase	**-2,4**	-
RNABJ9	AW916382	solute carrier family 15 (H+/peptide transporter), member 2	**-2**	1,2
RNABL90	AA819142	EST, selenium-binding protein	**-2,5**	-1,4

4 dpi				
RGIAM85	NM_012504	ATPase, Na+K+ transporting	-1,2	**-1,9**
**Immune system**				
1 dpi				
RGIAI44	AW140467	C10	**-1,6**	-
RGIBC16	BF281806	RT1 class Ib	**-2,1**	-1,1
**Growth factors and hormones**				
1 dpi				
RNABO23	AA899788	insulin-like growth factor II	**-1,7**	-1,6
RNABQ38	AA925112	EST, stromal cell-derived factor-1 gamma	**-2,6**	-1,5
**Miscellaneous**				
1 dpi				
RGIAJ86	L00091	angiotensinogen	**-2,2**	-1,1
RNABY86	AI145745	cpg2	**-1,7**	-1,7
RGIAK66	AW140528	carbonic anhydrase-related protein 2 precursor	**-1,6**	-1,3
RGIAL68	NM_008013	fibrinogen-like protein 2	**-2**	-1,3
RNABA70	BG380112	annexin A7	**-2,6**	-1,4
RNABY95	AI145705	EST, N-copine	**-1,8**	-
RGIBB48	AI171601	EST, synaptonemal complex protein 3	**-1,7**	1,1
RNACB85	AA964584	EST, oligodendrocyte-myelin glycoprotein precursor	**-1,7**	-
RNACE16	AI030090	EST, claudin-7	**-2,7**	-1,3
RGIBA74	AW141064	*hypothetical protein FLJ10134	**-1,9**	-
4 dpi				
RNABT1	AA957534	brain-enriched guanylate kinase-associated protein 1	-1,2	**-1,8**
RNABZ9	AI146115	smooth muscle-associated cell protein 8	-1,3	**-1,6**
**Unknown**				
1 dpi				
RGIAX45	AW142869	unknown	**-2,9**	-2,2
RGIBA58	AW142964	unknown	**-2,1**	-1
RNABC3	AW916600	unknown	**-2,3**	1,1
RGIBB66	AW144567	unknown	**-2**	-1,2
RGIAX65	AW142880	unknown	**-1,7**	-1,2
RGIAO71	AW142534	unknown	**-1,8**	-1
RGIAM79	AW142481	unknown	**-1,7**	-1,3
RGIBB18	AW142993	unknown	**-1,8**	-1
RNABG75	AW144173	unknown	**-1,7**	-1,3
RGIAZ93	AW142944	unknown	**-1,8**	-
RGIAF62	AW914464	unknown	**-1,6**	-
4 dpi				
RGIBA60	AW144560	unknown	-1,4	**-1,7**
